# Parental attitudes towards measles vaccination in the canton of Aargau, Switzerland: a latent class analysis

**DOI:** 10.1186/s12879-016-1747-0

**Published:** 2016-08-11

**Authors:** Carine Weiss, Daniel Schröpfer, Sonja Merten

**Affiliations:** 1 Swiss Tropical and Public Health Institute, Basel, Switzerland; 2 University of Basel, Basel, Switzerland; 3 Stadtärztlicher Dienst Zürich/Ambulatorium Kanonengasse, Zürich, Switzerland

**Keywords:** Measles immunisation, Parental attitudes towards vaccination, Latent class analysis

## Abstract

**Background:**

Despite the successes of routine national childhood vaccination programmes, measles remains a public health concern. The purpose of this paper is to investigate how patterns of parental attitudes are linked to the decision-making process for or against MMR vaccination. This exploratory study was designed to identify distinct patterns of attitudes towards or against measles vaccination through Latent Class Analysis (LCA) in a sub-sample of mothers living in the canton of Aargau in Switzerland.

**Methods:**

Parents of young children below 36 months of age were randomly selected through parents’ counsellors’ registries. Among other questions, respondents were asked to state their agreement in response to 14 belief statements regarding measles vaccination on a 5-point Likert scale. To identify groups of parents showing distinct patterns of attitudes and beliefs regarding measles vaccination, we used Latent Class Analysis (LCA).

**Results:**

The LCA showed three classes of parents with different attitudes and believes towards measles vaccination: The biggest group (class 1) are those having positive attitudes towards immunisation, followed by the second biggest group (class 2) which is characterised by having fearful attitudes and by showing uncertainty about immunisation. The third group (class 3) shows distinct patterns of critical attitudes against immunisation. Within this group over 90 % agree or totally agree that immunisation is an artificial intrusion into the natural immune system and therefore want to vaccinate their children only if necessary.

**Conclusion:**

We find that parents in the Canton Aargau who hesitate to vaccinate their children against measles, mumps and rubella show distinct opinions and attitudes. Health professionals should be aware of these perceptions to tailor their messages accordingly and positively influence these parents to vaccinate their children. Special attention needs to be given to those parents who are planning to vaccinate their children but are not following the national guidelines.

## Background

Although the measles vaccine has been part of routine national childhood vaccination programmes for more than 20 years, measles remains a public health concern. Large-scale outbreaks of vaccine preventable diseases in Europe and the US have re-emerged over the last decades and have affected the general population [1]. Switzerland faced two major measles outbreaks in 2006 and 2009 with more than 4400 reported measles cases including some hospitalisations and over 988 reported cases were reported between 2010 and 2013 [2, 3]. Switzerland had by far the highest incidence rate compared to other European countries [4–6]. Since 2005 population sensitisation has been strengthened resulting in a moderate increase of the coverage rate of two measles mumps and rubella (MMR) doses among 2-year old children to 83 % in 2011 [3]. This still remains below the recommended 95 % coverage of World Health Organisation. Moreover Switzerland shows high variation of MMR coverage between regions and cantons ranging from 50 to 93 % [3].

Following the sharp decline in the incidence of the majority of vaccine-preventable childhood diseases a number of parents emerged who perceive little risk associated with these infections. Instead, there is increasing fear that the risk from vaccination may outweigh the risk from natural infection, a reason for vaccination hesitancy. Consequently, a growing group of parents argue to better acquire immunity in a natural way [7–9]. Anti-vaccine movements have become more common and combined with a high proportion of unvaccinated children the current situation is of great public health concern. In response to the unsatisfactory vaccination coverage Switzerland developed a national strategy 2011–2015 aimed at the elimination of measles in the country [4].

Within the context of this strategy messages tended to focus on rational choice considerations, and point, for example, to the effectiveness of a vaccination to prevent long periods of sickness. Yet a parent’s decision to immunize a child is not based solely on the efficiency of a vaccine to prevent sickness, but also on emotions linked to particular ideologies about health and wellbeing [10]. An exploratory qualitative study conducted in 2010 in 4 cantons in Switzerland suggests that for some parents childhood diseases are thought to be part of a natural process of acquiring immunity and are not necessarily perceived as a threat to the health of the child. Parents argued against vaccination by saying that the child needs to acquire his own natural immunity and that immunisation is unnatural and superficial and therefore an artificial intrusion into the natural immune system [7, 11, 12]. Some parents also argued that the combined MMR vaccine “overloads” the immune system [13]; other parents may have had measles, mumps or rubella themselves when they were a child and did not perceive the disease as severe [14, 15]. Other reasons not to vaccinate a child varied from religious beliefs [16, 17] to lack of knowledge about vaccine-preventable diseases [18] to fears of side effects [19, 20]. Although considerable research has been devoted to emotion-related factors associated with immunisation status of the child, less attention has been paid to what kind of attitudes and belief systems drive parents not to vaccinate their children and whether specific attitude patterns are more likely to be associated with the vaccination status of the child.

The purpose of this paper is to investigate how patterns of parental attitudes are linked to the decision-making process for or against MMR vaccination. This exploratory study was designed to identify distinct patterns of attitudes towards or against measles vaccination through Latent Class Analysis [21] in a sub-sample of mothers living in the canton of Aargau in Switzerland.

## Methods

This article reports findings from a cross-sectional study in the canton of Aargau conducted in 2011. The canton had larger measles outbreaks in 2008–2009, and showed an average to high measles immunisation coverage of 89 % in 2011 [3]. Parents of young children below 36 months old were randomly selected through parents’ counsellors’ registries.

The questionnaire was informed by the concept of cultural epidemiology and addressed different dimensions of illness with a focus on subjective experience and perception of measles and measles vaccination [22–25]. The themes included in the questionnaire were derived from a qualitative study conducted some months before in the same target group [26]. More specifically, the questionnaire included parent-reported vaccination status of the youngest child, socio-demographic variables of parents and their children, parents’ knowledge of symptoms, disease experience, health seeking behaviour, risk perceptions, and affirmations on attitudes and opinions regarding measles vaccination. Fourteen statements illustrating parental attitudes and opinions were derived from the previously conducted qualitative study. Respondents were asked to state their agreement in response to the 14 belief statements on a 5-point Likert scale. The vaccination status of the child was based solely on the reported data of the parents and was not cross-validated with medical records.

The questionnaires were anonymously sent back by the parents. The data was double-entered into EpiInfo 3.5 and analysed with STATA SE 12.1.

To identify groups of parents showing distinct patterns of attitudes and beliefs which relate to measles vaccination we used Latent Class Analysis (LCA). This approach is based on the assumption that there are subgroups of parents (latent classes) within our sample, which are not directly observed but can be inferred from the observed attitudes of individuals [21]. All of the 14 above-mentioned statements on measles-related attitudes and beliefs were included in the latent class analysis in order to identify groups of parents with certain attitudes and beliefs towards measles immunisation. The number of classes was determined using the Bayesian Information Criterion (BIC). Each individual was then assigned to the class with the highest membership probability [21].

## Results

Out of 700 questionnaires sent to the canton Aargau, 101 were returned back to us as blank questionnaires by the parents’ counsellors, while 599 were forwarded to parents. Of those 599, 189 (32 %) were returned filled out. The questionnaire was predominantly filled out by mothers (98 %). The following analyses are based on the sample size of 189. The population characteristics are summarized in Table 1.

**Table 1:** Characteristics of survey participants (*N* = 189)

Age of respondent	% (*n*)
20–30	11.5 (21)
30–34	40.7 (75)
> 34	47.8 (88)
Marital status	
Married	87.0 (161)
Living together	7.57 (14)
Divorced	3.24 (6)
Single	1.62 (3)
Education	
Primary/secondary	4.9 (9)
High School (Matura)/ apprenticeship	53.5 (99)
University	40.5 (75)
Employment	
House wife	29.5 (55)
Employed	69.9 (130)
Residence	
Agglomeration	43.6 (78)
Rural	40.2 (72)
Urban	16.2 (29)

The LCA led to the identification of three latent classes characterized by distinct patterns of the 14 variables measuring parental attitudes. The groups are labelled according to their most pertinent attitudes (Table 2): positive, critical and fearful/uncertain attitudes.

**Table 2:** Results of three latent classes characterized by patterns of the 14 variables measuring parental attitudes through LCA (*N* = 175)

		Answered “Agree/Totally agree”		
Statements		Class 1 “positive attitudes” %	Class 2 “fearful/uncertain attitudes” %	Class 3 “critical attitudes” %
a	Measles vaccination may cause complications and I don’t want to put my child at risk.	18	25	94
b	I think it is better for my child if a vaccine can be swallowed then injected.	56	62	57
c	Vaccination is not compatible with homeopathy.	41	54	55
d	Immunisation is an artificial intrusion into the natural immune system and therefore I only want to vaccinate when it is really necessary.	15	25	90
e	In general little is known about the long-term effects of vaccines.	63	80	100
f	Measles isn’t dangerous and therefore vaccination is not really necessary.	0	32	64
g	In Switzerland in general immunisation is not very important as we have a high standard of living .	0	9	59
h	In Switzerland we can abstain from measles vaccination because we can treat complications of the vaccination or the measles itself.	0	40	87
i	Measles vaccination is unnecessary because complications related to the illness are rare.	5	46	81
j	I trust the advice of my paediatrician regarding if and when I should vaccinate.	96	99	71
k	I trust the advice of my family/ friends regarding if and when I should vaccinate.	45	64	50
l	I trust the advice of the Swiss federal office of public health (BAG) regarding immunisation.	89	81	17
m	Measles vaccination is unnecessary and only serves the pharmaceutical industries	1	10	89
n	Our government decides on vaccination recommendations independently of the pharmaceutical industries.	51	30	5
	*N*	101	49	25
Do you think in general immunisation is a reasonable method to prevent illness?				
Yes in general reasonable		65.3	36.7	12
It depends on the vaccination		34.7	59.2	48
Not very reasonable		0	0	16
Only exceptionally reasonable		0	4.1	16
Don’t know		0	0	8 *
In your opinion, how dangerous are measles for a child?				
Very dangerous		30.6	8.2	8
Somewhat dangerous		59.2	53.1	28
Not dangerous		1	4.1	32
Unsure		9.2	34.7	32 *
Do you think that measles vaccination is sensible for your child/ children?				
Yes		96	77.6	48
Not important		0	4.1	4
Only for certain people		1	14.3	12
Problematic		0	0	20
Don’t know		3	4.1	16 *
Do you plan to vaccinate your youngest child against measles?				
Already immunised		47.5	34.7	32
Planned		49.5	51	24
Don’t want to immunise		0	4.1	28
Unsure		3	10.2	16 *
Total *N*		101	49	25

*p = 0.000

The biggest group (class 1) are those having positive attitudes towards immunisation, in particular measles vaccination (*n* = 101). They state clearly that measles is somewhat dangerous and vaccination is important (statement f: 0 %), that immunisation in Switzerland is important despite our high living standards (statement g: 0 %) and that we cannot reject measles immunisation as we can treat complications easily (statement h: 0 %). This group also agrees highly with the recommendations of the paediatrician (statement j: 96 %) as well as with the recommendation from the Swiss federal office of public health (statement l: 89 %).

The second biggest group (class 2) is characterised by having fearful attitudes and by expressing uncertainty about immunisation (*n* = 49). Class 2 responded less distinct to the majority of statements. A third of class 2 respondents agree that measles are not dangerous and therefore vaccination is unnecessary (statement f: 32 %). They also responded more positively that in Switzerland measles vaccination is not necessary as complications can be easily treated (statement h: 40 %) and that complications in case of illness are rather rare (statement i: 46 %). This group also strongly follows the recommendations of the paediatrician (statement j: 99 %), the Swiss federal office of public health (statement l: 81 %) and the advice of friends and family members if and when to vaccinate (statement k: 64 %).

The third group (class 3) shows distinct patterns of critical attitudes against immunisation (*n* = 25). Within this group over 90 % agree or totally agree that immunisation is an artificial intrusion into the natural immune system and therefore want to vaccinate their children only if necessary (statement d: 90 %). They fear more often side effects of the vaccination and prefer not to put their healthy child at risk (statement a: 94 %). They also state that in Switzerland measles immunisation is unnecessary as complications can easily be treated (statement h: 87 %) and that complications in case of illness are rare (statement i: 81 %). They also agree more often than other groups that measles vaccination is unnecessary and only serves the pharma industry (statement m: 89 %). There were no significant associations of the latent class membership with age, parity, or educational level.

Table 2 shows significant differences in the opinion and risk perceptions about measles vaccination between the three groups of parents designated by the LCA.

In general parents with positive attitudes (class 1) towards vaccination responded significantly more often that vaccination is a reasonable method to prevent illness (65.3 %), compared to those who have some doubts towards immunisation (class 2) (36.7 %) and who have critical attitudes (class 3) (12 %) (*p* = 0.000).

Parents with positive attitudes (class 1) and with uncertain attitudes (class 2) significantly more often cited that measles is somewhat or very dangerous (89.8 vs. 61.3 %) compared to the parents with critical attitudes (class 2) (36 %) (*p* = 0.000).

Half of the parents who are less inclined to vaccinate their children (class 2) reported that measles vaccination is reasonable for their children (48 %), compared to those who are in favour of measles vaccination (class 1) (96 %) and those who are doubtful (class 2) (77.6 %) (*p* = 0.000).

Parents were asked whether they immunised their youngest child against measles or whether they plan to do so. Those with critical attitudes (class 3) significantly more often cited not vaccinating their children (28 %) compared to the other two groups (class 1: 0 %; class 3: 4.1 %) (*p* = 0.000).

Table 3 shows knowledge of measles symptoms according to the three groups of parents. There are no significant differences between the three groups and knowledge of symptoms.

**Table 3:** Knowledge of Measles symptoms

Knowledge of measles symptoms:	“positive attitudes” %	“fearful/ uncertain attitudes” %	“critical attitudes” %
Koplik’s spots			
Very often	92.9	85.7	76
Sometimes	5.1	10.2	20
Rare	0	2	0
Don’t know	2	2	4
Total *N* (ns)	99	49	25
Fever			
Very often	82.8	69.4	76
Sometimes	16.2	24.5	20
Rare	0	2	0
Don’t know	1	4.1	4
Total *N* (ns)	99	49	25
Headache			
Very often	21.5	20.9	16.7
Sometimes	38.7	25.6	41.7
Rare	16.1	20.9	16.7
Never	3.2	4.7	4.2
Don’t know	20.4	27.9	20.8
Total *N* (ns)	93	43	24
Sore throat			
Very often	17.6	19	13
Sometimes	27.5	21.4	26.1
Rare	18.7	16.7	17.4
Never	8.8	9.5	8.7
Don’t know	27.5	33.3	34.8
Total *N* (ns)	91	42	23
Pneumonia			
Very often	3.4	0	0
Sometimes	12.4	21.1	8.7
Rare	38.2	23.7	47.8
Never	18	21.1	8.7
Don’t know	28.1	34.2	34.8
Total *N* (ns)	89	38	23
Unconsciousness			
Sometimes	4.5	5.4	4.5
Rare	40.4	35.1	27.3
Never	22.5	18.9	22.7
Don’t know	32.6	40.5	45.5
Total *N* (ns)	89	37	22
Vomiting			
Very often	11	0	0
Sometimes	17.6	15.4	30.4
Rare	34.1	30.8	26.1
Never	9.9	15.4	4.3
Don’t know	27.5	38.5	39.1
Total *N* (ns)	91	39	23
Convulsion			
Very often	2.2	0	4.3
Sometimes	13.2	5.3	21.7
Rare	39.6	34.2	30.4
Never	13.2	15.8	13
Don’t know	31.9	44.7	30.4
Total *N* (ns)	91	38	23

Note: *ns* not significant

In general knowledge level about measles symptoms are rather low especially for less pronounced symptoms such as headache, sore throat, pneumonia and unconsciousness.

## Discussion

Recent measles outbreaks, stagnating measles vaccination coverage and the emergence of anti-vaccine movements are of public health concern in Switzerland. Although the vast majority of parents continue to have their children vaccinated, there has always been a minority of people opposed to vaccination. The aim of this exploratory study was to investigate the perceptions, attitudes and opinions of parents about measles vaccination and the disease. The findings from this exploratory study offer original insights into parents’ perceptions and attitudes about measles immunisation and immunisation behaviours which we anticipate will contribute to the refinement of a larger population-based study in Switzerland.

Our exploratory study shows that LCA is a useful method to classify parents according to their opinions and attitudes for or against measles immunisation [21]. The LCA produced three groups of parents with distinct patterns of attitudes: class 1 was the biggest group and was the group with “positive attitudes” toward measles vaccination This group reported significantly more often to have vaccinated their children against measles. Class 3 showed critical attitudes, considered vaccination against measles as unnatural and unnecessary in a developed country such as Switzerland, and was less inclined to vaccinate their children. Parents of class 2 were uncertain and fearful about the need to vaccinate against measles, but were inclined to follow the official recommendations to vaccinate their children.

The results demonstrate that the mothers seemed confident that vaccination protects their children against measles, mumps, and rubella, an attitude which reflects the high MMR vaccination coverage of the canton of Aargau. To most of the respondents, who tended to be well educated, mostly employed and older than 30 years of age, this confidence constituted sufficient reason to vaccinate their children. The majority of the mothers knew the predominant symptom of measles to be Koplik’s spots. Good knowledge about the disease and the vaccination has been positively associated with higher vaccination status in adults [18]. In this study knowledge about measles does not seem to influence the decision making of whether to vaccinate or not. There seem to be other mechanisms which shape parental thoughts about measles vaccination.

There is a considerable number of respondents that believe that immunization does not provide sufficient benefit to justify the risk of vaccination (“class 2 and 3”). Most of the parents see vaccination as a personal decision, which takes the particularities of their children’s immune system into account [7, 8]. This is reflected in the statement a) “Measles vaccination may cause complications and I don’t want to put my child at risk” and d) “Immunisation is an artificial intrusion into the natural immune system and therefore I only want to vaccinate when it is really necessary” which is predominately agreed with by class 3 parents (“having critical attitudes”).

The class 3 group also does not seem to mind taking the risk that their children naturally contracts a disease than ‘causing’ their children damage through vaccination [11, 27, 28]. This is reflected in the statements f) “measles isn’t dangerous and therefore vaccination not really necessary” and i) “measles vaccination is unnecessary because complications are rare in case of an illness”. In a qualitative study Gross et al. show that in Switzerland, relatively highly educated mothers with critical attitudes towards measles vaccination tended to believe that acquiring measles naturally is better for the development of the immune system of their children as compared to vaccination [29]. This quantitative study, which was designed as a complementary study to Gross et al., validates findings on the parental concept of naturalness and “natural” functioning of the human immune system. Our analysis confirms that vaccination hesitancy or resistance may not merely be caused by distrust in the health system, the pharmaceutical industry and government agencies. In this sub-population it seems that beliefs and opinions are more crucial than health system factors (statement j): “I trust the advice of my paediatrician if and when to vaccinate”. Instead, hesitancy seems to be at least partly driven by a trend towards “natural”, “sustainable” and “healthy” living.

Avoiding overloading the immune system and not interfering with the “nature” of their children’s immune system seem to be of high importance to a growing number of parents. Those who refuse to be vaccinated themselves or do not want their children to be vaccinated are more often seeking complementary and alternative medicine care compared to non-users [30, 31]. However, homeopathy for example did not influence the decision making process considerably in our study, which might be due to Aargau being a canton with high MMR coverage.

Gross et al. point out that their findings gear towards a personalized and patient-centred approach with regards to a child’s vaccination plan [29]. This is also reflected in our findings. The group of female parents who expressed fearful or uncertain attitudes towards vaccination (class 2) highly agree to follow the paediatrician’s advice (statement j) as well as the advice of the Swiss federal office of public health (statement l). This group shows hesitancy to vaccinate their children due to uncertainties about the effect of the vaccine. Targeting this group with individualised messages respecting their concern and/or a less rigorous immunisation plan may positively influence vaccination coverage or decisions to vaccinate.

Studies in other industrialized countries show that the parental decision making process around vaccination against preventable childhood diseases is multi-faceted [10, 11]. Decision making processes of parents were shown to be guided either by the risk posed by the disease [32], the risk of the vaccine aimed at preventing the disease [27, 28, 33] or the perceived strength of the immune system to cope with the disease if contracted naturally [11, 20]. Similar trends can be observed in this study, especially in the attitudes of parents critical of measles vaccination.

This study has several limitations. The response rate was 32 % and families who agreed to participate might have been more receptive to preventive actions than the general population. This means that for example in class 3, where currently 28 % are not vaccinating, the rate of non-vaccination in this population group might in fact be higher. A study conducted in Sweden examining the reasons why a small minority of Swedish parents choose not to vaccinate their children reported that parents who are willing to vaccinate their children were more likely to fill out the questionnaire [32]. We do not have information about non-responders in our study. In addition, this questionnaire was almost uniquely filled out by the mothers of children although fathers were approached as well. It would be interesting to investigate whether there is a gender difference between parents’ beliefs and attitudes towards vaccination.

In addition we encountered reluctance of health professionals to support the recruitment of parents in other cantons, as it was initially planned to recruit from different sites. Alfredson et al. experienced negative reactions from health professionals who feared conflicts with parents with unvaccinated children, similar to our study [32].

## Conclusions

To counteract the fears and misperceptions associated with vaccine campaigns, the research community and ministries of health/ federal office of public health need to be proactive with regard to continued vaccine education, guiding public perception with rigorous scientific research on vaccine safety and emphasizing the importance of vaccination in preventing unwanted and potentially lethal infectious diseases. In conclusion, we find that parents who hesitate to vaccinate their children against measles, mumps and rubella have distinct opinions and attitudes while emphasizing healthy living. Health professionals should be aware of these perceptions in order to positively influence these parents to vaccinate their children and to tailor vaccination messages accordingly [32]. Special emphasis needs to be given to those parents who are planning to vaccinate their children but are not following the national guidelines [14, 32]. This group may fall behind schedule or forget to vaccinate their children and thus create a threat for other children in case of a measles outbreak. There might be a need to shift thinking towards a more flexible approach on the vaccine schedule following up those parents more closely [29].

## Abbreviations

CI, confidence interval; LCA, latent class analysis.
